# Exploring a Region on Chromosome 8p23.1 Displaying Positive Selection Signals in Brazilian Admixed Populations: Additional Insights Into Predisposition to Obesity and Related Disorders

**DOI:** 10.3389/fgene.2021.636542

**Published:** 2021-03-25

**Authors:** Rodrigo Secolin, Marina C. Gonsales, Cristiane S. Rocha, Michel Naslavsky, Luiz De Marco, Maria A. C. Bicalho, Vinicius L. Vazquez, Mayana Zatz, Wilson A. Silva, Iscia Lopes-Cendes

**Affiliations:** ^1^Department of Medical Genetics and Genomic Medicine, Brazilian Institute of Neuroscience and Neurotechnology (BRAINN), University of Campinas – UNICAMP, Campinas, Brazil; ^2^Departament of Genetics and Evolutive Biology, Human Genome and Stem Cell Research Center, Institute of Bioscience, University of São Paulo (USP), São Paulo, Brazil; ^3^Department of Surgery, Federal University of Minas Gerais (UFMG), Belo Horizonte, Brazil; ^4^Department of Clinical Medicine, Federal University of Minas Gerais (UFMG), Belo Horizonte, Brazil; ^5^Molecular Oncology Research Center (CPOM) – Barretos Cancer Hospital, Barretos, Brazil; ^6^Department of Genetics, Ribeirão Preto Medical School, University of São Paulo at Ribeirão Preto (USP), Ribeirão Preto, Brazil

**Keywords:** population genomics, Latin American populations, complex diseases, risk stratification, genomic medicine, precision medicine

## Abstract

We recently reported a deviation of local ancestry on the chromosome (ch) 8p23.1, which led to positive selection signals in a Brazilian population sample. The deviation suggested that the genetic variability of candidate genes located on ch 8p23.1 may have been evolutionarily advantageous in the early stages of the admixture process. In the present work, we aim to extend the previous work by studying additional Brazilian admixed individuals and examining DNA sequencing data from the ch 8p23.1 candidate region. Thus, we inferred the local ancestry of 125 exomes from individuals born in five towns within the Southeast region of Brazil (São Paulo, Campinas, Barretos, and Ribeirão Preto located in the state of São Paulo and Belo Horizonte, the capital of the state of Minas Gerais), and compared to data from two public Brazilian reference genomic databases, BIPMed and ABraOM, and with information from the 1000 Genomes Project phase 3 and gnomAD databases. Our results revealed that ancestry is similar among individuals born in the five Brazilian towns assessed; however, an increased proportion of sub-Saharan African ancestry was observed in individuals from Belo Horizonte. In addition, individuals from the five towns considered, as well as those from the ABRAOM dataset, had the same overrepresentation of Native-American ancestry on the ch 8p23.1 locus that was previously reported for the BIPMed reference sample. Sequencing analysis of ch 8p23.1 revealed the presence of 442 non-synonymous variants, including frameshift, inframe deletion, start loss, stop gain, stop loss, and splicing site variants, which occurred in 24 genes. Among these genes, 13 were associated with obesity, type II diabetes, lipid levels, and waist circumference (*PRAG1*, *MFHAS1*, *PPP1R3B*, *TNKS*, *MSRA*, *PRSS55*, *RP1L1*, *PINX1*, *MTMR9*, *FAM167A*, *BLK*, *GATA4*, and *CTSB*). These results strengthen the hypothesis that a set of variants located on ch 8p23.1 that result from positive selection during early admixture events may influence obesity-related disease predisposition in admixed individuals of the Brazilian population. Furthermore, we present evidence that the exploration of local ancestry deviation in admixed individuals may provide information with the potential to be translated into health care improvement.

## Introduction

Admixture between different continental populations generates mosaic chromosomes comprised of genomic segments with different ancestry, which is defined as local ancestry (Seldin et al., 2011). As a result, admixed populations may present marked differences in local ancestry patterns (Browning et al., 2016; Deng et al., 2016; Martin et al., 2017; Secolin et al., 2019). These differences may impact disease incidence and genetic risk prediction across populations (Myles et al., 2008; Moonesinghe et al., 2012; Martin et al., 2017). Thus, enhancing our knowledge of the effect of local ancestry is crucial for the development of adequate precision health programs in admixed populations (Aronson and Rehm, 2015; Hindorff et al., 2018).

The Brazilian population was formed via an admixture process comprised mostly of European, sub-Saharan African, and Native-American population ancestry. In terms of global ancestry inference, studies have shown a predominance of European ancestry, followed by sub-Saharan African and Native-American (Kehdy et al., 2015; Lima-Costa et al., 2015; Rodrigues de Moura et al., 2015). Furthermore, a recent study about local ancestry inferences reported that the Native-American component predominated on the chromosome (ch) 8p23.1 due to positive selection (Secolin et al., 2019) (3). Ch 8p23.1 has undergone inversion events stratified across continental populations (Salm et al., 2012), which may influence the recombination landscape (Alves et al., 2014).

Interestingly, the ch 8p23.1 region found to be under positive selection in the Brazilian population has been reported to contain genes previously associated with type 2 diabetes and overweight/obesity in admixed Americans (Dunn et al., 2006; Flores et al., 2016). Indeed, studies taking admixture into account have shown that type 2 diabetes, insulin secretion, body mass index, obesity, and adiposity are the main clinical phenotypes associated with metabolic disorders (Dunn et al., 2006; Hayes et al., 2013; Goetz et al., 2014; Flores et al., 2016; Mehta et al., 2017). Thus, we hypothesize that variants in candidate genes located on ch 8p23.1 could have provided an evolutionary advantage in a restrictive diet environment in the early stages of the Brazilian admixture. However, in the present high caloric diet environment, this genetic variability can result in an increased number of obesity-related traits in admixed Brazilian individuals.

Therefore, our objective was to expand our knowledge of the effects of admixture by describing the genetic variability of ch 8p23.1 from admixed Brazilian exomes compared with global populations. To achieve our aim, we first extended our study to additional admixed exomes from other southeastern Brazil towns. Second, we identified and analyzed sequencing variants identified in the candidate region of ch 8p23.1.

## Materials and Methods

### Subjects

We evaluated 257 individuals from BIPMed (Rocha et al., 2020), 609 from ABraOM (Naslavsky et al., 2017), and 88 additional exomes from individuals born in the following towns within southeastern Brazil: Barretos (*N* = 30); Ribeirão Preto (*N* = 30), located in the state of São Paulo; and Belo Horizonte (*N* = 28), the capital of the state of Minas Gerais (Supplementary Figure 1). Among individuals included in BIPMed, the birthplace of 193 individuals were included; thus, we were able to extract 21 individuals born in São Paulo city and 37 from Campinas to increase the power of regional comparisons. No information regarding place of birth was obtained from the ABraOM dataset. Permission to use raw, anonymized data from BIPMed and ABraOM public databases and raw, anonymized data associated with the 88 exomes of individuals from Barretos, Ribeirão Preto, and Belo Horizonte was obtained. This study was approved by the University of Campinas’s Research Ethics Committee (UNICAMP, Campinas, São Paulo, Brazil). All methods were performed following relevant guidelines and regulations.

### Exome Processing

Exome data were stored in variant call format (VCF) files created using the GRCh37 assembly. We used PLINK 1.9 (Purcell et al., 2007) software to convert VCF to PLINK files, variant and individual filtering, and data merging (Anderson et al., 2010). First, we removed ambiguous variants (with G/C or A/T alleles) from VCFs associated with each town, BIPMed, and ABraOM. Next, we merged all Brazilian VCFs, maintaining only biallelic variants, autosomal variants, variants in Hardy–Weinberg equilibrium (Anderson et al., 2010), and removal of missing data (> 10%). These filters were used only to analyze population structure and local ancestry and were removed in the analysis to identify variants in the candidate region at ch 8p23.1.

We evaluated the heterozygosity rate of each individual to search for inbreeding (low heterozygosity rate) or sample contamination (high heterozygosity rate) (Anderson et al., 2010), and individuals with a heterozygosity rate higher or lower than three standard deviations (SDs) from the mean were removed. We also removed individuals with genomic relatedness matrix estimations higher than 0.125, which is the expected genomic relatedness of third-degree relatives (Anderson et al., 2010). The genomic relatedness matrix estimation used a greedy algorithm implemented using the PLINK 1.9 software to maximize the sample size.

After genotype and individual filtering, a total of 893 exomes and 661,617 variants remained in the Brazilian datasets analyzed. We merged this dataset with the 1000 Genome project data phase 3 (1KGP) (1000 Genomes Project Consortium et al., 2015) and removed SNPs with a minor allele frequency (MAF) < 0.01. As a result, 225,997 variants for local ancestry inference were used. We also removed variants in linkage disequilibrium (LD) from the MAF-filtered dataset (parameters: window size = 50 SNPs; shift step = 5 SNPs; and r2 = 0.5) (Anderson et al., 2010), which left 127,172 SNPs for an investigation of population structure.

### Population Structure

To evaluate whether our Brazilian sample (BRS) presents a geographical substructure based on birthplace, we performed the analysis of molecular variance (AMOVA) using the poppr.amova package in R software (Excoffier et al., 1992), which compares the genetic distance among birthplace/town groups based on a set of 10,000 random SNPs across the genome. In addition, we compared the BRS data classified by birthplace to the 1KGP dataset via principal component analysis (PCA) using PLINK v1.9 software to evaluate the presence of population-based outliers in the BRS dataset.

### Local Ancestry Inference and Positive Selection Test

We phased SNPs without LD pruning using the SHAPEIT2 v2.r387 software with default parameters (O’Connell et al., 2014). After phasing, we converted the output data from SHAPEIT2 to input files required by RFMix v.1.5.4 software (Maples et al., 2013) using a pipeline previously reported^1^ (Martin et al., 2017).

Previous studies showed that using Peruvian individuals from the 1KGP with a high degree of Native-American ancestry as a Native-American reference produced the same result as using Native-American indigenous individuals (Secolin et al., 2019). Therefore, we inferred the local ancestry of 23 Peruvian individuals who possessed a > 0.95 proportion of Native-American ancestry (NAT) (Secolin et al., 2019), 23 random Europeans (EUR), and 23 random sub-Saharan Africans from the 1KGP (AFR). The size sample of ancestry references was selected based on the 23 NAT to avoid biases due to unbalanced reference panel sizes of ancestry references, according to the RFMix v.1.5.4. Manual (Maples et al., 2013). We ran RFMix in PopPhased mode with a minimum window size of 0.2 cM, using one EM iteration and node size 5. The reference panel was maintained after the initial inference step, and forward-backward probabilities were saved. We analyzed the proportion of EUR, AFR, and NAT ancestry in the BRS dataset for each variant across the genome using in-house-developed R scripts (Secolin et al., 2019), and results were plotted using the man package in R software (Turner, 2014). In order to evaluate the presence of ch 8p32.1 inversions, we performed an inversion inference using the invClust package in R software (Cáceres and González, 2015), as performed in our previous work (Secolin et al., 2019). Since we have individuals that overlap the previous paper, we decided to compare the inversion inference between the SNP array data (Secolin et al., 2019) and the exome data from the same individuals to evaluate whether the inversion analysis generated a perfect match.

We tested our exome sample for positive selection by the same approach used previously (Patin et al., 2017). Briefly, this approach combines the results of five neutrality statistics (intrapopulation absolute integrated haplotype scores (| iHS|, | ΔiHH|) (Voight et al., 2006; Sabeti et al., 2007), interpopulation integrated haplotype score (| ΔiHH_*derived*_|) (Grossman et al., 2010), interpopulation extended haplotype homozygosity (XP-EHH) (Sabeti et al., 2007), and population branch statistics (PBS) (Yi et al., 2010) based on Hudson’s Fst (Bhatia et al., 2013) in a single Fisher combined score (FCS) (Deschamps et al., 2016). The variants with values of FCS higher than 99% of the SNP FCS values across the genome (i.e., the 1% highest FCS values) were defined as outliers. Then, we split the genome into 100-variants blocks. Finally, we estimated the proportion of outliers within each block. If a block presents a proportion of outliers higher than the 99.5th percentile (the highest 0.5%), it was defined as a region under positive selection.

### Analysis of Chromosome 8p23.1

We extracted the ch 8p23.1 region (8092025–11859740 bp) (Secolin et al., 2019) from the VCF file of each sample using vcftools (Danecek et al., 2011). Variant consequences from each gene region were annotated using the ANNOVAR software (version 2019Oct24) (Wang et al., 2010) with the following flags: -other info (to include our sample AF); -one transcript; -buildver hg19; -remove; -protocol refGene, gnomad211_exome, ALL.sites.2015_08,EUR.sites.2015_ 08,AFR.sites.2015_08,AMR.sites.2015_08,EAS.sites.2015_08,SAS. sites.2015_08,dbnsfp35a; -operation g,f,f,f,f,f,f,f,f; and -nastring.

We included the allele frequency (AF) information from African/African-American (AFR/AFA), Latino/admixed American (LAT/AMR), East Asian (EAS), non-Finish European (NFE), and South Asian (SAS) populations from the gnomAD exome dataset (Karczewski et al., 2020); sub-Saharan African (AFR), Europeans (EUR), admixed Americans (AMR), East Asians (EAS), and South Asians (SAS) from 1KGP, which are publicly available in ANNOVAR resource data. In addition, we annotated variants that were not identified via ANNOVAR using the Variant Effect Prediction (VEP) algorithm (McLaren et al., 2016) with the following parameters: a buffer_size 500; –canonical; –distance 5000; –regulatory; –species homo_sapiens; –symbol.

To predict the impact of non-synonymous variants identified on protein function, we analyzed the information provided by the use of 12 algorithms, which included PolyPhen2 (Adzhubei et al., 2013), Sort Intolerant from Tolerant (SIFT) (Sim et al., 2012), MutationTaster (Schwarz et al., 2010), PROVEAN (Choi et al., 2012), Combined Annotation Dependent Depletion (CADD) (Rentzsch et al., 2019), MutPred^2^. Functional Analysis through Hidden Markov Models (FATHMM) (Shihab et al., 2015), PhD-SNPg (Capriotti and Fariselli, 2017), Condel (González-Pérez and López-Bigas, 2011), PANTHER (Mi et al., 2013), Align Grantham Variation/Grantham Difference score (GVGD) (Tavtigian et al., 2006), and SNPs&GO (Calabrese et al., 2009). For Align-GVGD, variants graded higher than C35 were classified as deleterious. For MutPred2, variants with a score higher than 0.5 were considered pathogenic. For all other algorithms, we used default classifications.

Associated trait information for genes located on ch 8p23.1 was accessed from the NHGRI-EBI GWAS Catalog (Buniello et al., 2019) on October 30, 2020, and results were complemented by a search of the PubMed^®^ database.

## Results

### Population Structure

**FIGURE 1:**
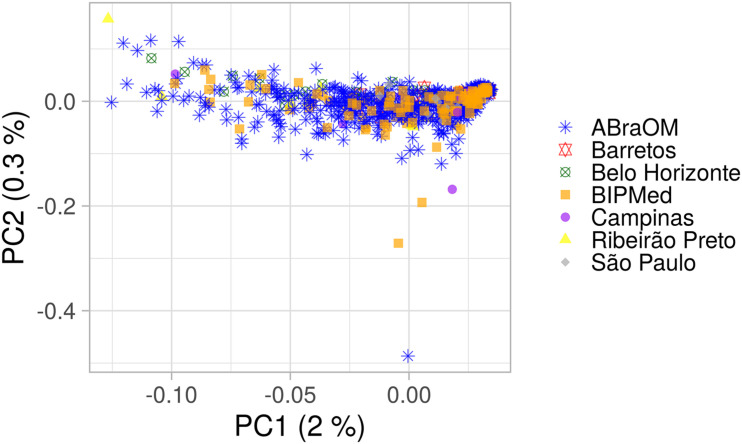
PCA plot of the BRS sample. The *x*-axis and *y*-axis show the first and second principal components (PC1 and PC2) and their respective percentage variability. Each point represents one individual, and each shape and color indicate a birthplace or database. Only information from individuals with a known place of birth was included.

AMOVA results obtained using 10,000 random SNPs showed that 99.21% of observed variation occurred within groups and 0.79% occurred among groups (total φ-statistics = 0.0079; *p* = 0.0001), indicating the absence of a population substructure, which is consistent with the lack of clusters observed in the PCA of the BRS sample that was based on birthplace (Figure 1). In addition, the global PCA of the 1KGP dataset (Supplementary Figure 2) showed that our sample consisted of a mixture of European, sub-Saharan African, and Native-American/East Asian individuals, similar to other admixed American populations. However, the population was distributed mainly between Europeans and sub-Saharans rather than Native-Americans, consistent with previous studies (Ruiz-Linares et al., 2014; Kehdy et al., 2015; Secolin et al., 2019; Rocha et al., 2020).

### Local Ancestry Inference

**FIGURE 2:**
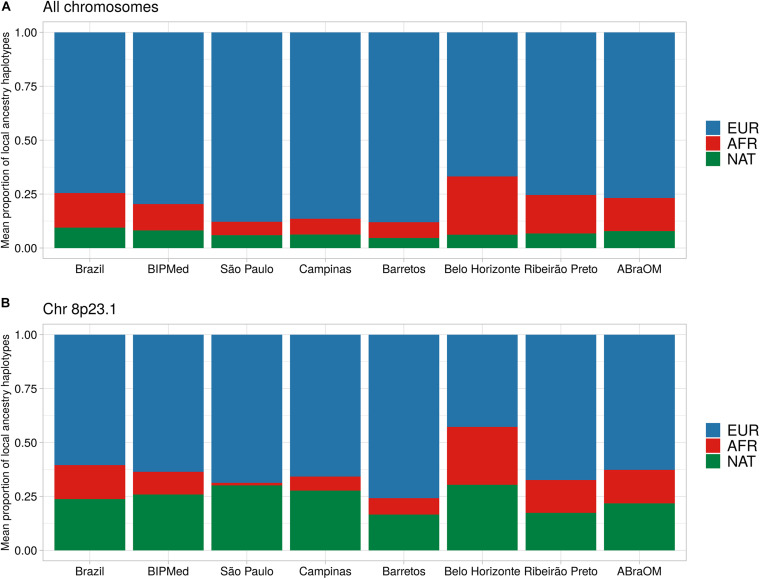
Barplot of the mean proportion of local ancestry haplotypes. Each bar represents one Brazilian sample. **(A)** Mean local ancestry haplotypes across all exomes. **(B)** Mean local ancestry haplotypes on chr8p23.1. EUR, European ancestry component; AFR, African ancestry component; NAT, Native-American ancestry component.

The proportion of the BRS sample, which included individuals born in the different towns and two public datasets, had an average local ancestry proportion for its EUR component of 74.6% (*SD* = 1.4%). The proportion of the sample that comprised the AFR component was 16.0% (*SD* = 1.1%), and the NAT component was 9.4% (*SD* = 1.1%) (Figure 2A and Supplementary Figure 3). We observed differences in EUR and AFR ancestry proportions among towns, with the Belo Horizonte population containing the lowest EUR component (mean = 66.8%; *SD* = 6.0%), and the highest AFR component (mean = 26.9%; *SD* = 5.6%). São Paulo, in contrast, had the greatest proportion of EUR ancestry (mean = 87.8%; *SD* = 5.7%) and the lowest AFR proportion (mean = 6.2%; *SD* = 4.1%). The NAT component of the sample remained constant among individuals from the different towns and the two public Brazilian databases, ranging from a mean of 4.6% (*SD* = 2.8%) in Barretos to 8.2% (*SD* = 1.3%) in the BIPMed sample (Figure 2A). Our assessment revealed a decreased EUR component on ch 8p23.1, and an elevated NAT in individuals born in Campinas, São Paulo, Barretos, and Belo Horizonte, as well as in those included in the ABraOM dataset (Figure 2B and Supplementary Figures 3–9).

We inferred the inversion events on ch 8p23.1 from the exome data by the same approach used in our previous report (Secolin et al., 2019), with the invClust package in R (Cáceres and González, 2015). The results showed that 48.9% of the inferred inversions in the exome data matched the results previously obtained with the SNP-array dataset of the BIPMed sample used in our previous work (Secolin et al., 2019; Supplementary Table 1).

We tested for positive selection in the exome dataset using the Fisher combined scores (FCS). FCS, which includes PBS tests in the calculation, this is the same approach used in our previous work (Secolin et al., 2019). However, the results did not recapture the same positive selection signal on ch 8p23.1 previously observed (Secolin et al., 2019) (Supplementary Figures 3–10).

### Analysis of Chromosome 8p23.1

We found 17,536 variants within ch 8p23.1. We focused on the following variants with the potential to impact gene function: 414 non-synonymous variants, ten frameshifts, eight inframe deletions, one start loss, five stop gains, one stop loss, and five splicing sites. The variants affected 24 genes and two open reading frames (Supplementary Data Sheet 1). Among these variants, 355 were also found in gnomAD and/or 1KGP databases, and 44 such variants were determined to be common with an alternative allele frequency (AAF) > 0.01 in the BRS dataset but rare (AAF < 0.01) in gnomAD and 1KGP (Table 1). Also, we identified nine common variants (AAF > 0.01) among the 89 variants exclusive to the Brazilian population. The AF comparison of these 89 variants separated by Brazilian cities and datasets showed that the *RP1L1* gene in the ABraOM database contained the largest number of exclusive Brazilian variants (Supplementary Figure 11).

**TABLE 1:** Distribution of genetic variants found in the candidate region of ch 8p23.1, classified according to allele frequencies (AF) observed in the different datasets studied.

AF distribution (*p* = 2.2e^–16^)*	Common in the Brazilian sample	Rare in the Brazilin sample	Total
Common in gnomAD and/or 1KGP	69 (19.4%)	3 (0.9%)	72 (20.3%)
Rare in gnomAD and/or 1KGP	44 (12.4%)	239 (67.3%)	283 (79.7%)
Total	113 (31.8%)	242 (68.2%)	355 (100%)

*Calculated using Fisher’s exact test. 1KGP, 1000 Genome Project phase 3.

**FIGURE 3:**
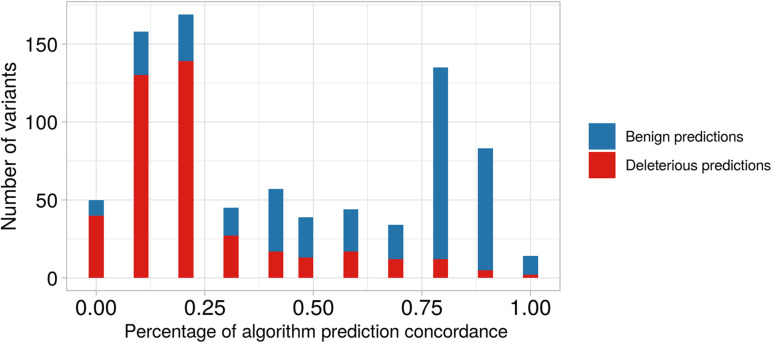
Barplot of predictive algorithm concordance between benign versus deleterious variant predictions. On the *x*-axis, we show the percentage of concordance among the different algorithms. On the *y*-axis, we show the number of predicted variants. For example, the second bar represents the number of variants predicted with low concordance among different algorithms (∼12.5%), and we observe that the number predicted to be deleterious is higher than that predicted to be benign. In contrast, the tenth bar shows predictions with high concordance among algorithms (∼87.5%), and we observe that the number of predicted benign variants is higher than predicted deleterious variants.

We observed that 374 of the 414 non-synonymous variants, in genes located at ch 8p23.1, were classified as deleterious via *in silico* prediction of at least one algorithm (Supplementary Data Sheet 2), and 19 of these were predicted to be pathogenic with an 80% concordance among the different algorithms; these were present in five genes *PRSS55*, *RP1L1*, *SOX7*, *GATA4*, and *CTSB*. As shown in Figure 3 and Supplementary Table 2, we found 167 non-synonymous variants predicted to be benign by at least one algorithm; these were present in 16 different genes. Also, there were 309 variants predicted to be deleterious and 50 variants predicted to be benign when considering less than 20% concordance among the algorithms.

Interestingly, 140 of the variants predicted to be benign by at least one algorithm (140/167) were found in 13 genes, which were previously associated with metabolic phenotypes such as type 1 diabetes mellitus (*T1DM*), type 2 diabetes mellitus (*T2DM*), obesity, insulin resistance, body mass index (BMI), body fat distribution, waist circumference, and diet measurement (*MFHAS1*, *ERI1*, *TNKS*, *PRSS55*, *RP1L1*, *PINX1*, *XKR6*, *FAM167A*, *BLK*, *GATA4*, and *CTSB* genes), Table 2. Also, 45 of these variants were located in the following eight genes with an AAF > 0.01, considering the BRSs and gnomAD/1KGP databases: *MFHAS1*, *ERI1*, *PRSS55*, *RP1L1*, *PINX1*, *FAM167A*, *GATA4*, and *CTSB* (Supplementary Data Sheet 2).

**TABLE 2:** Genes associated with obesity-related traits that localize to the candidate region on ch 8p23.1 and are found to contain genetic variants in the Brazilian datasets analyzed in the present work.

Gene	Variant count		Associated trait	Population analyzed	References
	
	Benign	Deleterious			
*CLDN23*	3	–	–	–	–
*MFHAS1*	4	–	T2DM; cooked vegetable consumption; fish- and plant-related diet	European; African American; Hispanic; Asians; East Asian; South Asian;	Niarchou et al., 2020; Vujkovic et al., 2020
*ERI1*	1	–	Obesity; BMI; body fat distribution	European; Asian; Hispanic; Native-American; Oceanian	Pulit et al., 2019; Rask-Andersen et al., 2019; Schlauch et al., 2020
*TNKS*	2	–	T2DM; BMI; Early-onset extreme obesity	European; French and German groups	Scherag et al., 2010; Xue et al., 2018; Wang et al., 2019
*PRSS55*	9	3	Waist circumference	Hispanic obesity children	Comuzzie et al., 2012
*RP1L1*	107	2	Waist circumference	Waist circumference	Comuzzie et al., 2012
*C8orf74*	7	–	–	–	–
*SOX7*	–	2	–	–	–
*PINX1*	8	–	T2DM; Lipid levels	European, South Asian, East Asian, African	Willer et al., 2013; Xue et al., 2018; Wang et al., 2019
*XKR6*	1	–	T2DM; BMI; body fat distribution; raw vegetable consumption; processed meat consumption; fish- and plant-related diet	European	Mahajan et al., 2018; Pulit et al., 2019; Rask-Andersen et al., 2019; Niarchou et al., 2020
*SLC35G5*	13	–	–	–	–
*FAM167A*	4	–	T2DM	African American; Caribbean	Divers et al., 2017
*BLK*	1	–	T2DM	European	Borowiec et al., 2009
*GATA4*	1	2	T1DM; Neonatal and Childhood-Onset diabetes; fruit consumption, processed meat consumption	European	Sartori et al., 2014; Shaw-Smith et al., 2014; Niarchou et al., 2020
*NEIL2*	2	–	–	–	–
*CTSB*	2	1	Obesity; visceral obesity in T2DM; non-alcoholic fatty liver disease	Danes; Finnish; European	Peltola et al., 2006; Andreasen et al., 2009; Chalasani et al., 2010
*DEFB136*	2	-	-	-	-

*T1DM, type 1 diabetes mellitus; T2DM, type 2 diabetes mellitus; BMI, body mass index.*

## Discussion

The sequencing analysis of ch 8p23.1 performed in the current study revealed the presence of 442 non-synonymous variants, including frameshift, inframe deletion, start loss, stop gain, stop loss, and splicing site variants, which occurred in 24 genes and two open reading frames. Among the genes, 13 were associated with obesity, type II diabetes, lipid levels, and waist circumference (*PRAG1*, *MFHAS1*, *PPP1R3B*, *TNKS*, *MSRA*, *PRSS55*, *RP1L1*, *PINX1*, *MTMR9*, *FAM167A*, *BLK*, *GATA4*, and *CTSB*).

The inversion event on ch 8p23.1 generated a large haplotype, which was able to be traced through continental populations globally (Salm et al., 2012). It presented us with an opportunity to investigate how admixture events and evolutionary processes have affected variability within non-inverted and inverted haplotypes in admixed populations. Previously, two independent studies reported local ancestry deviation on ch 8p23.1 in admixed American populations, likely due to inversion events (Guan, 2014; Secolin et al., 2019). Furthermore, our own work using SNP-array data demonstrated that the proportion of non-inverted haplotypes inherited from Native-Americans is higher than those inherited from Europeans in admixed Brazilian individuals (Secolin et al., 2019). Here, we replicated these findings using exome datasets in populations originating from an extended geographic region in the southeastern region of Brazil. Besides, since we evaluated individuals with unknown information regarding the presence of obesity-related disorders, our study is not biased toward a specific phenotype, and it is suitable to assess the genetic variability of the candidate region on ch 8p23.1.

We observed that the results from the inversion inference on ch 8p23.1 obtained in the present work, using the exome data, did not completely match that resulted from the analysis using the SNP-array dataset (Secolin et al., 2019). However, it is noteworthy that the invClust package was developed to be used with SNP-array data, and to our knowledge, there is no reference to its use with exome datasets. Thus, it is possible that inferences of chromosomal inversions using exome data may not be accurate with the invClust package. As pointed out in our previous work (Secolin et al., 2019), it is not likely that inversion bias would influence the high NAT proportions observed in the sample. However, we agree that this is a limitation of our current work. Further analysis, in which inverted and non-inverted genotypes are unequivocally identified, would help evaluate the distribution of the inflation in NAT ancestry in inverted and non-inverted genotypes.

There is evidence that the deviation towards Native-American ancestry on ch 8p23.1 could be due to positive selection events after the Brazilian admixture (Secolin et al., 2019). Indeed, previous studies suggested that Native-American ancestry was admixed early in the European colonization in Brazil (approximately 18 to 16 generations ago) and was followed by the posterior depletion of NAT (Kehdy et al., 2015). This early admixture could have catalyzed positive selection events among the first admixed Brazilian individuals. Although environmental causes that drove this positive selection remain unknown, studies had identified variants associated with type 2 diabetes mellitus, insulin secretion, body mass index, obesity, and adiposity, when admixture was considered (Dunn et al., 2006; Hayes et al., 2013; Goetz et al., 2014; Flores et al., 2016; Mehta et al., 2017). Therefore, the large number of genes located on ch 8p23.1 related to diet and metabolic traits suggest that positive selection may have occurred due to the restrictive diet environment and severe famine periods in early admixed Brazilian individuals (Davis, 2001).

In the present work, our results did not recapture the same positive selection signal detected previously (Secolin et al., 2019) (Supplementary Figures 3–10). However, since FCS has only been used with whole-genome sequencing (Deschamps et al., 2016; Patin et al., 2017) and SNP-array datasets (Secolin et al., 2019), we believe that the decrease in genetic variability present in exome data may render FCS less suitable for this type of analysis.

Increasing fat and glucose storage could increase body fat, glucose storage, and obesity-related diseases in individuals who eat a fat and glucose-rich diet today; findings consistent with previous association studies (Pulit et al., 2019; Rask-Andersen et al., 2019; Schlauch et al., 2020). Indeed, we identified 89 variants with the potential to impact gene function that were found exclusively in the admixed Brazilian sample. Unfortunately, we cannot define the correct phase for the allele variants and the ancestry block by the RFMix algorithm. However, we know that the 89 variants are in the region, presenting 60.69% of EUR ancestry proportion, followed by 15.47% of AFR and 23.83% of NAT ancestry proportions. The comparison of these proportions with the average EUR ancestry proportion among Brazilian genomes genomes (74.6%), AFR (16.0%), and NAT (9.4%) suggests that these variants present exclusively in the Brazilian samples could, most likely, be the main contributors to the signals of selection identified, and are possibly influencing obesity-related phenotypes. Therefore, we consider the region of ch 8p23.1 a hotspot for genetic variants that predispose individuals to obesity disorders. It may be useful, as a first strategy, to concentrate efforts on studying effects of non-synonymous variants identified within the 13 candidate genes of the region, *PRAG1*, *MFHAS1*, *PPP1R3B*, *TNKS*, *MSRA*, *PRSS55*, *RP1L1*, *PINX1*, *MTMR9*, *FAM167A*, *BLK*, *GATA4*, and *CTSB*. It may also be useful to expand genetic studies to include patients with obesity-related phenotypes and studying the expression levels of candidate genes in relevant tissue may also give additional clues regarding their roles in disease-related phenotypes.

In Table 2, we present the list of 17 genes that have been linked to diet patterns in large association studies and are located in the candidate region on ch 8p23.1. Seven of these large studies included Hispanic, Native-American, and Caribbean populations (Comuzzie et al., 2012; Divers et al., 2017; Pulit et al., 2019; Rask-Andersen et al., 2019; Niarchou et al., 2020; Schlauch et al., 2020; Vujkovic et al., 2020), and three contained association signals in the *ERI1* gene (Pulit et al., 2019; Rask-Andersen et al., 2019; Schlauch et al., 2020), which is located within the region and was determined to possess the greatest degree of positive selection in admixed Brazilians in our previous work (Secolin et al., 2019).

Finally, it is also important to study the non-synonymous variants identified in the candidate genes on ch 8p23 and predicted to be benign. These variants were identified in 11 of the candidate genes listed in Table 2. Currently, we cannot exclude the possibility that even though these variants are not predicted to affect protein function individually, they may contribute to a polygenic phenotype.

Furthermore, when considering a polygenic phenotype, one aspect that we should take into account is the presence of epistatic interactions. Thus, we could argue that an increase in the frequency of the genes of NAT ancestry on ch 8p23.1 could be due to the breakup of negative epistatic interactions among genes on other regions from NAT genomes and the genes on ch 8p23.1, which are currently coupled with AFR and EUR ancestry tracts, and could lead to increased fitness. We count the number of AFR-NAT-AFR, EUR-NAT-EUR, and NAT-NAT-NAT haplotypes, including ch 8p23.1 and adjacent regions (approximately 3.7Mb upstream and downstream ch 8p23.1). However, we did not observe an overrepresentation of AFR-NAT-AFR (*n* = 32) or EUR-NAT-EUR (*n* = 98) ancestry haplotypes compared to NAT-NAT-NAT haplotypes (*n* = 118). Therefore, our results suggest that interactions among EUR or AFR ancestry genes in adjacent regions on ch 8p23.1 with the NAT ancestry core seems not to be enough to boost negative or positive selection in our sample. However, gene interactions can occur among genes on ch 8p23.1 and genes on other regions of the genome, and further studies should be performed to clarify this issue.

## Conclusion

We successfully replicated previous results that identified local ancestry deviation on ch 8p23.1, which seems to have occurred in populations from the southeastern region of Brazil, including the states of São Paulo and Minas Gerais. Thus, the candidate region on ch 8p23.1 emerges as a hotspot for obesity-related genes in admixed Brazilians, which should be further explored. In particular, the information presented here could be used in the future to support risk stratification and implement personalized public health policies and preventive medical treatments.

## Data Availability Statement

The datasets presented in this study can be found in online repositories have been included in the Supplementary Material. The names of the repository/repositories and accession number(s) can be found in the article/Supplementary Material.

## Ethics Statement

The studies involving human participants were reviewed and approved by Comit de tica da Universidade Estadual de Campinas. The patients/participants provided their written informed consent to participate in this study.

## Author Contributions

RS created the study design, conceptualized the work, and performed data acquisition and analysis. MG performed the *in silico* prediction analysis. CR participated in BIPMed data acquisition. MN and MZ participated in the ABraOM data acquisition. LD and MB participated in the Belo Horizonte data acquisition and determined sample information. VV aided with the Barretos data acquisition and provided sample information. WS aided in the Ribeirão Preto data acquisition and provided sample information. IL-C conceptualized the work and served as the principal investigator. All authors reviewed and approved the final version of the manuscript.

## Conflict of Interest

The authors declare that the research was conducted in the absence of any commercial or financial relationships that could be construed as a potential conflict of interest.

## Footnotes

1 https://github.com/armartin/ancestry_pipeline

2 http://mutpred.mutdb.org
