# Supplementary material for: Exploring a Region on Chromosome 8p23.1 Displaying Positive Selection Signals in Brazilian Admixed Populations: Additional Insights Into Predisposition to Obesity and Related Disorders
Source: Front Genet. 2021 Mar 25;12:636542. doi: 10.3389/fgene.2021.636542 (PMC8027303; doi:10.3389/fgene.2021.636542)
Supplement: Supplementary file 3 [file Presentation_1.pdf]

Supplementary Material

Exploring a region on chromosome 8p23.1 displaying positive selection signals in Brazilian admixed populations: additional insights into predisposition to obesity and related disorders

Rodrigo Secolin, Marina C. Gonsales, Cristiane S. Rocha, Michel Naslavsky, Luiz De Marco, Maria A.C. Bicalho, Vinicius L. Vazquez, Mayana Zatz, Wilson A. Silva, Iscia Lopes-Cendes

The datasets analyzed in this study can be found in the EVA repository/PRJEB39251 (https://www.ebi.ac.uk/eva/?eva-study=PRJEB39251) , BIPMed (www.bipmed.org) , and ABraOM site (http://abraom.ib.usp.br/).

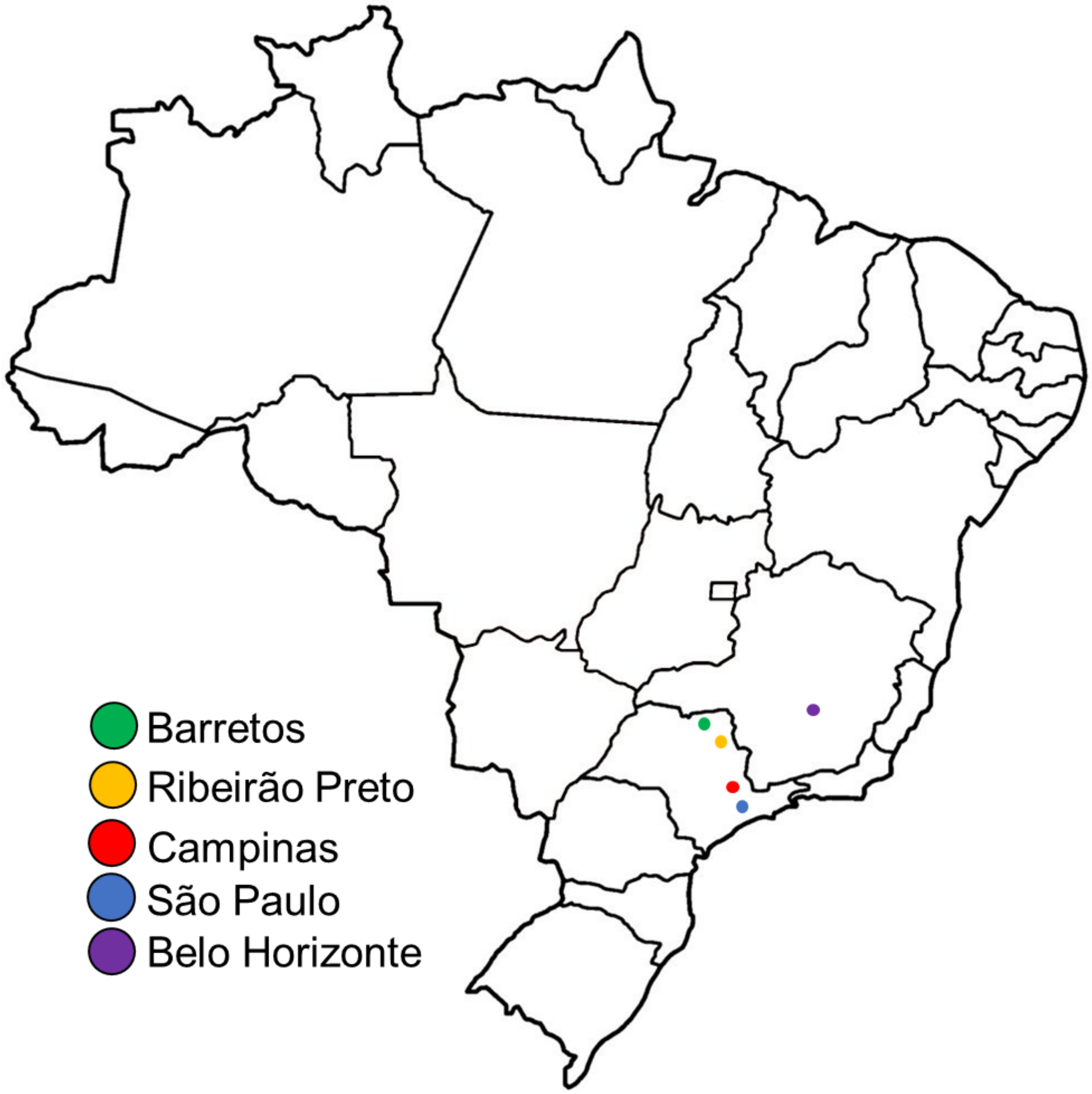

Supplementary Fig. 1. Map of Brazil separated by Federative States, indicating the geographic distribution of the towns from which the Brazilian samples were collected. BIPMed samples were collected in Campinas and ABraOM samples were collected in São Paulo city.

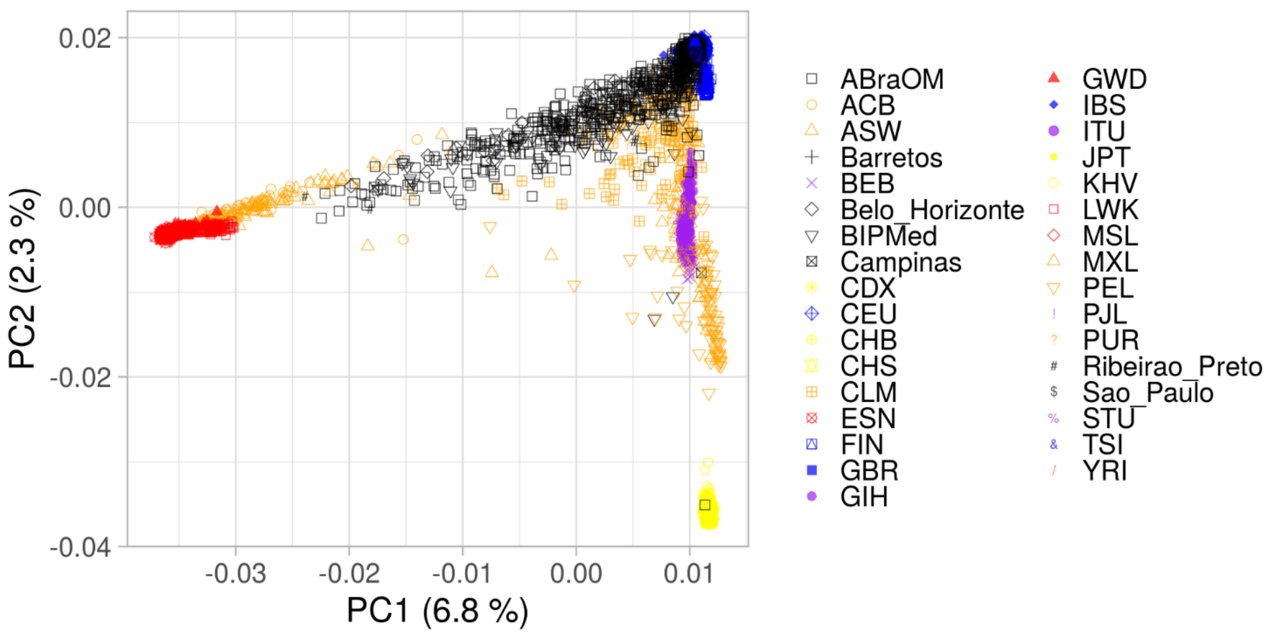

Supplementary Figure 2. PCA plot showing the Brazilian samples in black, and 1KGP populations. Each symbol represents a population, and colors represent each continental population, as follows: the sub-Saharan African population is shown in red; the European population is shown in blue; the East Asian population is shown in yellow; the South Asian population is shown in purple, and the admixed American population is shown in orange.

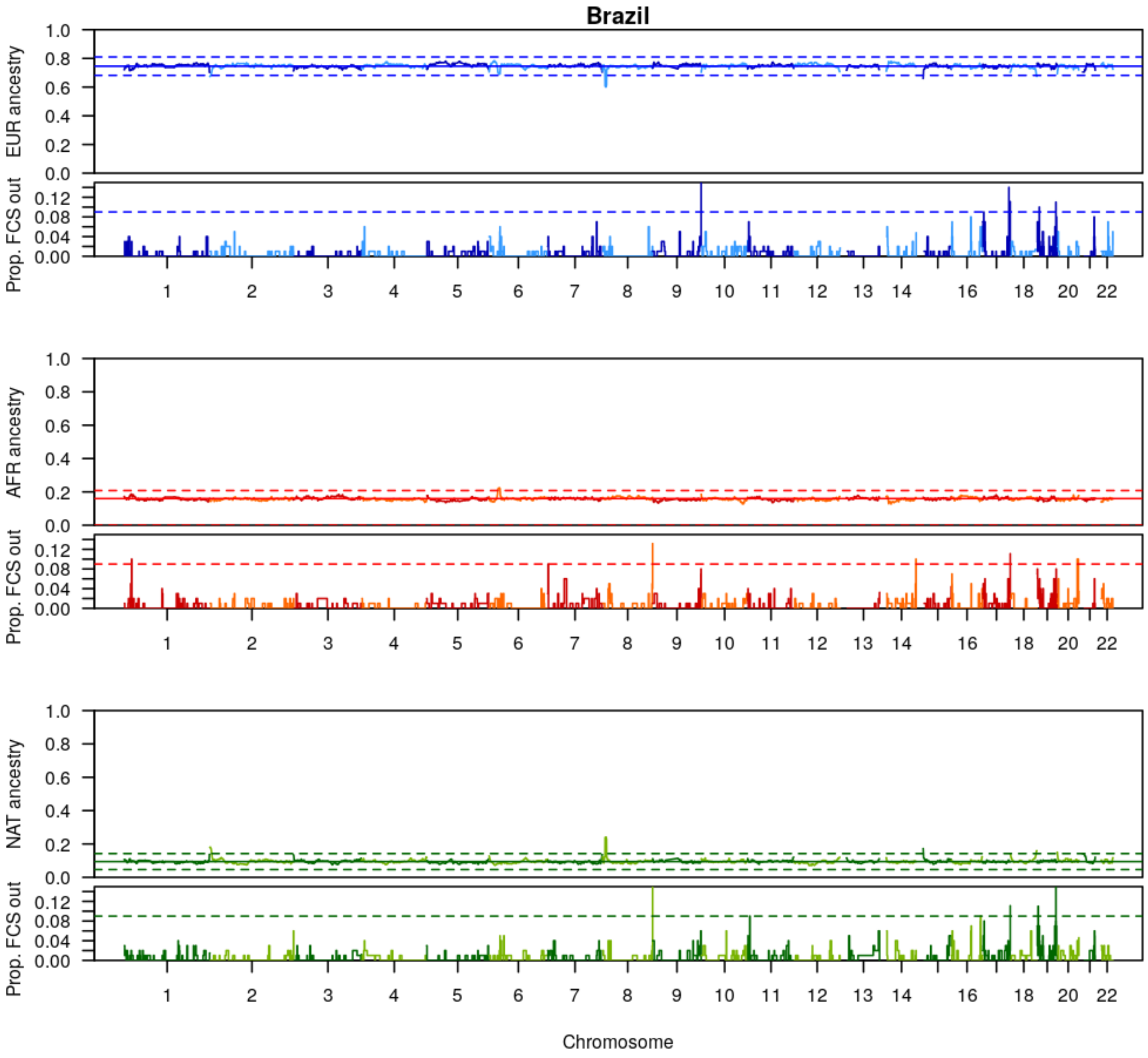

Supplementary Figure 3. Local ancestry inference and signals of positive selection based on Secolin et al., 2019, for the Brazil exome sample, including the five cities considered and ABraOM and BIPMed samples. Each chromosome is shown on the x-axis, and the proportions of European (EUR; grades of blue), sub-Saharan African (AFR; grades of red), and Native-American ancestries (NAT, grades of green) are indicated on the y-axis. Each dashed line indicates a 4.42 SD threshold. The signal of positive selection shows the proportion of FCS outliers (Prop. FCS out) in the y-axis, following the same colour pattern for each ancestry component. Each dashed line indicates the 99.5th percentile thresholds.

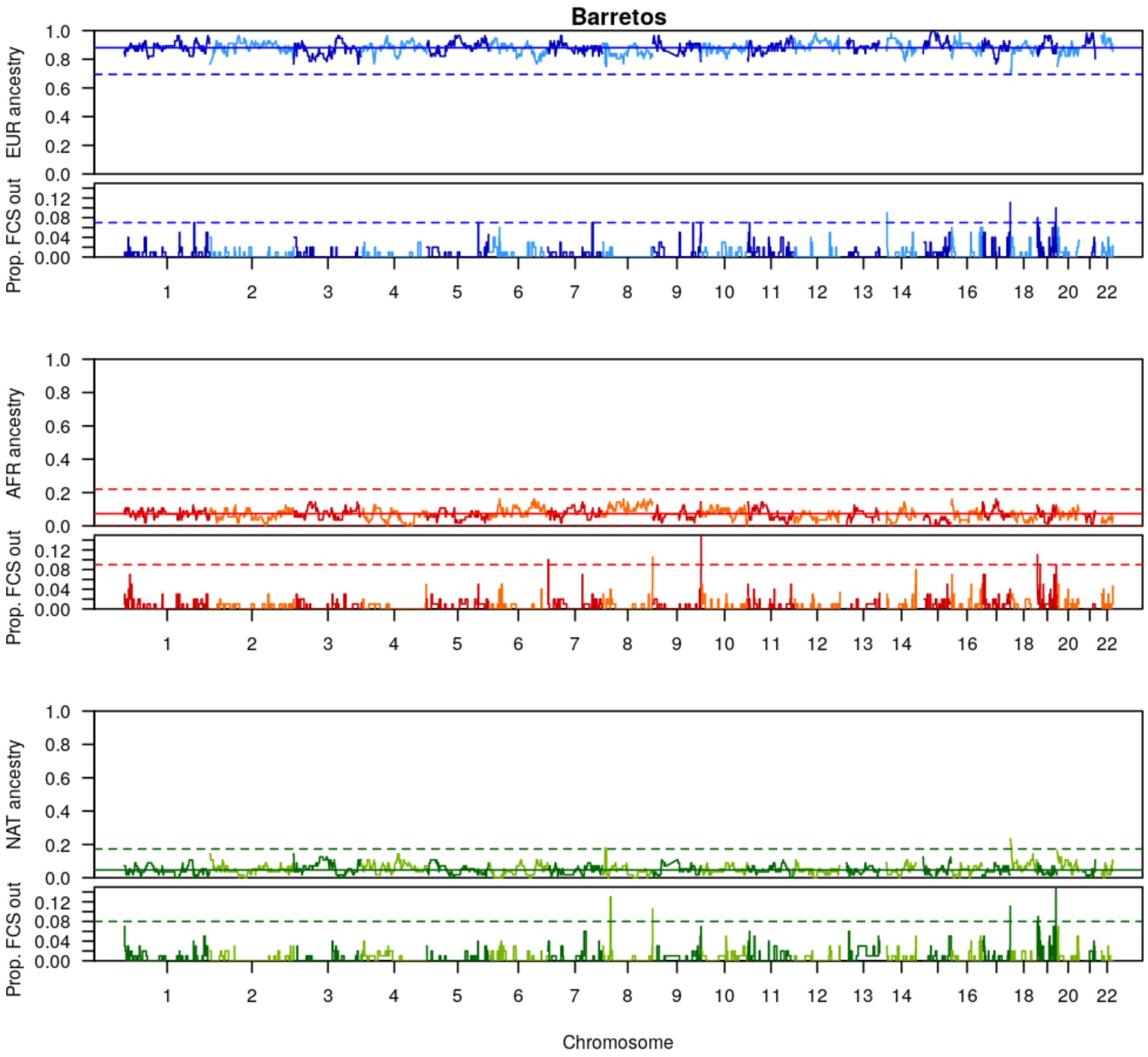

Supplementary Figure 4. Local ancestry inference and signals of positive selection based on Secolin et al., 2019, for Barretos exome sample. Each chromosome is shown on the x-axis, and the proportions of European (EUR; grades of blue), sub-Saharan African (AFR; grades of red), and Native-American ancestries (NAT, grades of green) are indicated on the y-axis. Each dashed line indicates a 4.42 SD threshold. The signal of positive selection shows the proportion of FCS outliers (Prop. FCS out) in the y-axis, following the same colour pattern for each ancestry component. Each dashed line indicates the 99.5th percentile thresholds.

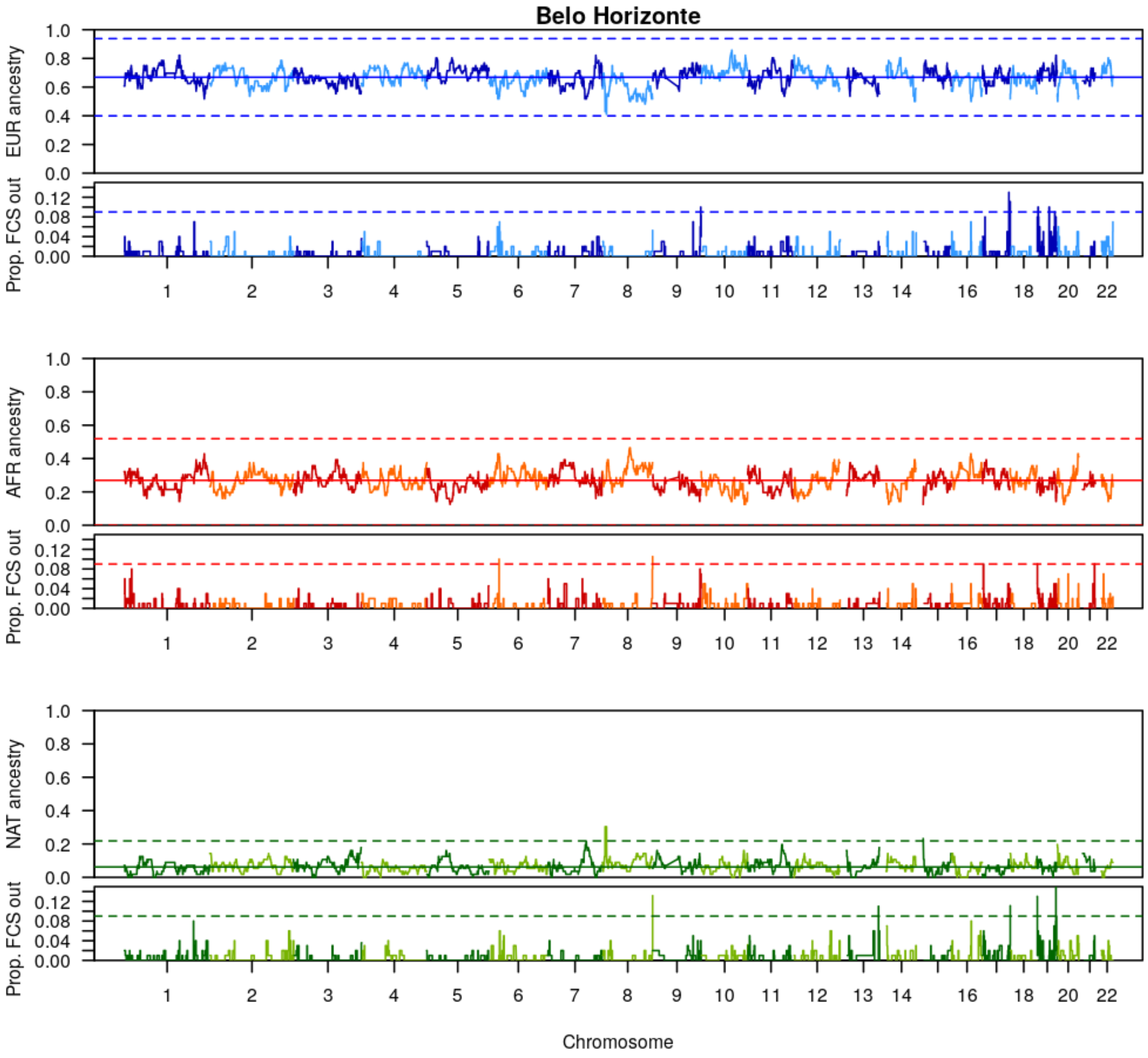

Supplementary Figure 5. Local ancestry inference and signals of positive selection based on Secolin et al., 2019, for Belo Horizonte exome sample. Each chromosome is shown on the x-axis, and the proportions of European (EUR; grades of blue), sub-Saharan African (AFR; grades of red), and Native-American ancestries (NAT, grades of green) are indicated on the y-axis. Each dashed line indicates a 4.42 SD threshold. The signal of positive selection shows the proportion of FCS outliers (Prop. FCS out) in the y-axis, following the same colour pattern for each ancestry component. Each dashed line indicates the 99.5th percentile thresholds.

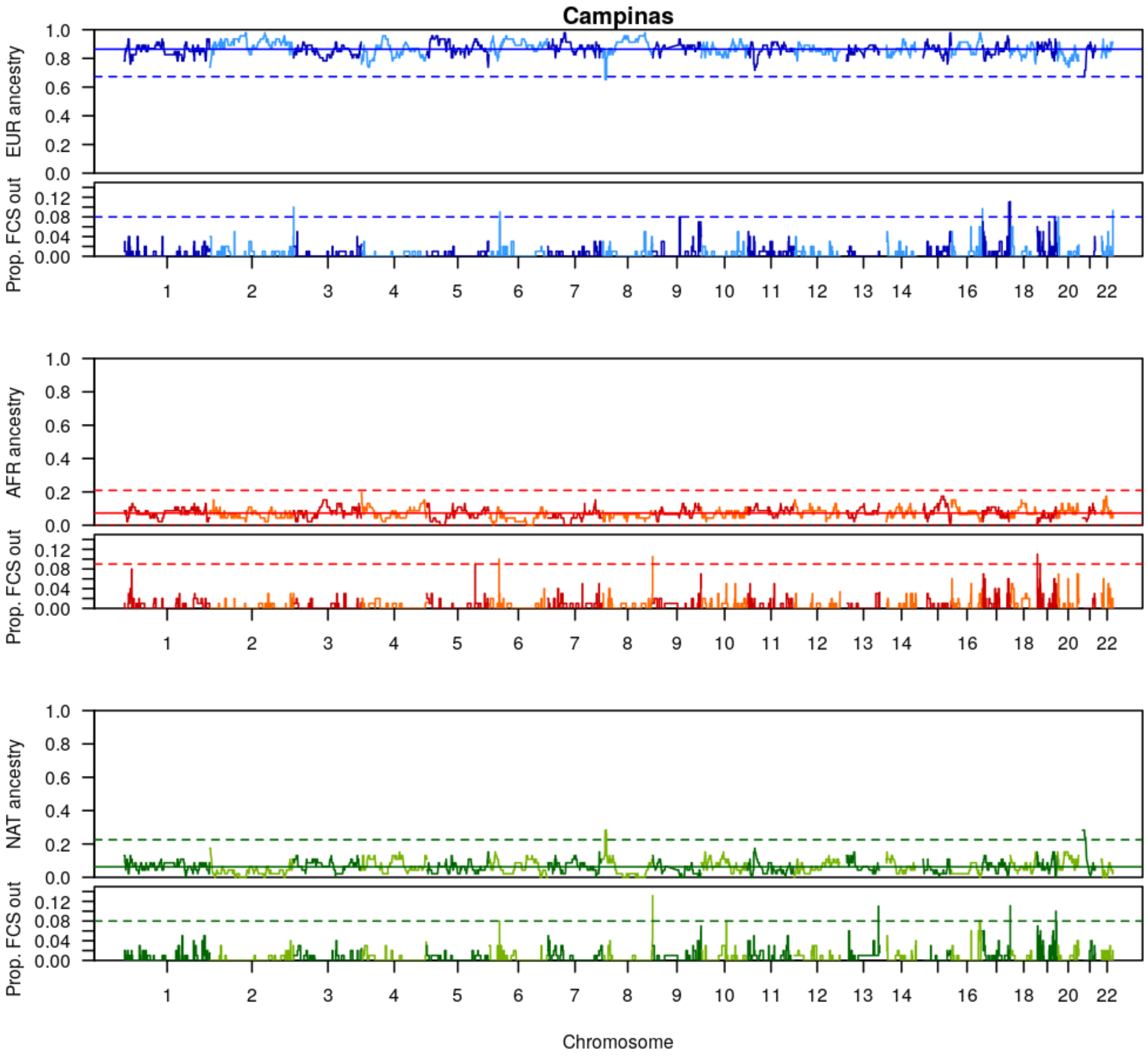

Supplementary Figure 6. Local ancestry inference and signals of positive selection based on Secolin et al., 2019, for Campinas exome sample. Each chromosome is shown on the x-axis, and the proportions of European (EUR; grades of blue), sub-Saharan African (AFR; grades of red), and Native-American ancestries (NAT, grades of green) are indicated on the y-axis. Each dashed line indicates a 4.42 SD threshold. The signal of positive selection shows the proportion of FCS outliers (Prop. FCS out) in the y-axis, following the same colour pattern for each ancestry component. Each dashed line indicates the 99.5th percentile thresholds.

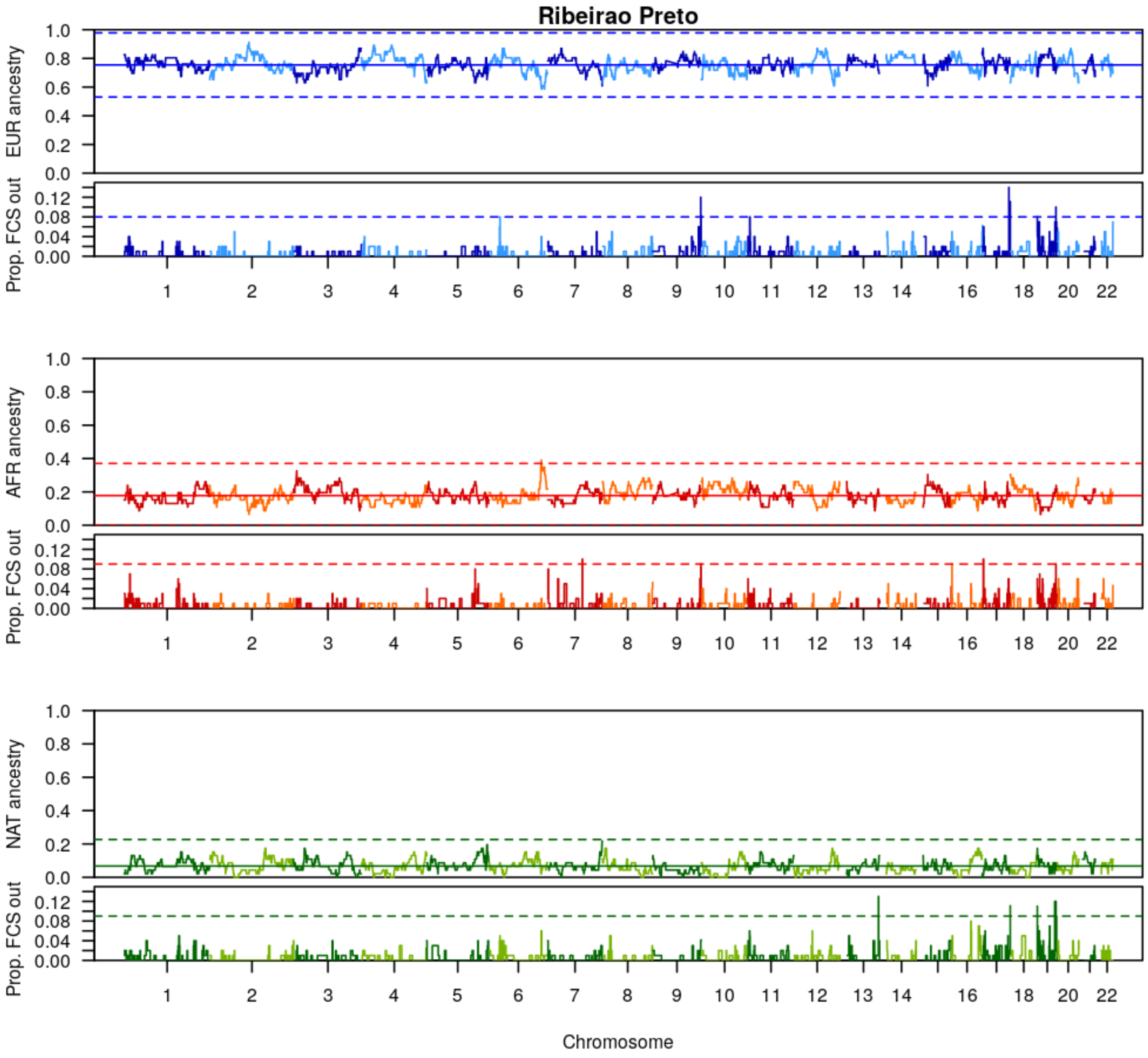

Supplementary Figure 7. Local ancestry inference and signals of positive selection based on Secolin et al., 2019, for Ribeirão Preto exome sample. Each chromosome is shown on the x-axis, and the proportions of European (EUR; grades of blue), sub-Saharan African (AFR; grades of red), and Native-American ancestries (NAT, grades of green) are indicated on the y-axis. Each dashed line indicates a 4.42 SD threshold. The signal of positive selection shows the proportion of FCS outliers (Prop. FCS out) in the y-axis, following the same colour pattern for each ancestry component. Each dashed line indicates the 99.5th percentile thresholds.

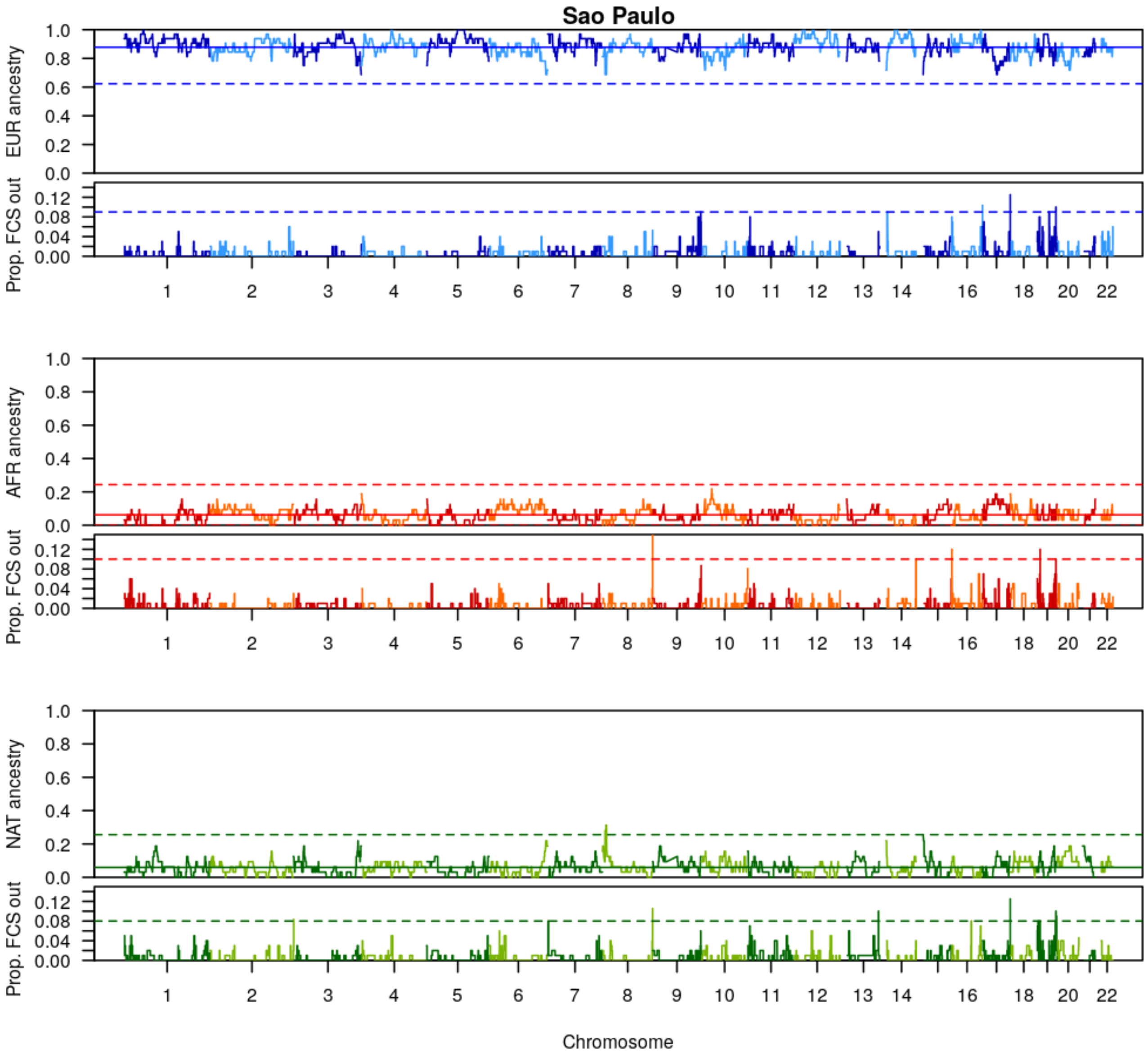

Supplementary Figure 8. Local ancestry inference and signals of positive selection based on Secolin et al., 2019, for Sao Paulo exome sample. Each chromosome is shown on the x-axis, and the proportions of European (EUR; grades of blue), sub-Saharan African (AFR; grades of red), and Native-American ancestries (NAT, grades of green) are indicated on the y-axis. Each dashed line indicates a 4.42 SD threshold. The signal of positive selection shows the proportion of FCS outliers (Prop. FCS out) in the y-axis, following the same colour pattern for each ancestry component. Each dashed line indicates the 99.5th percentile thresholds.

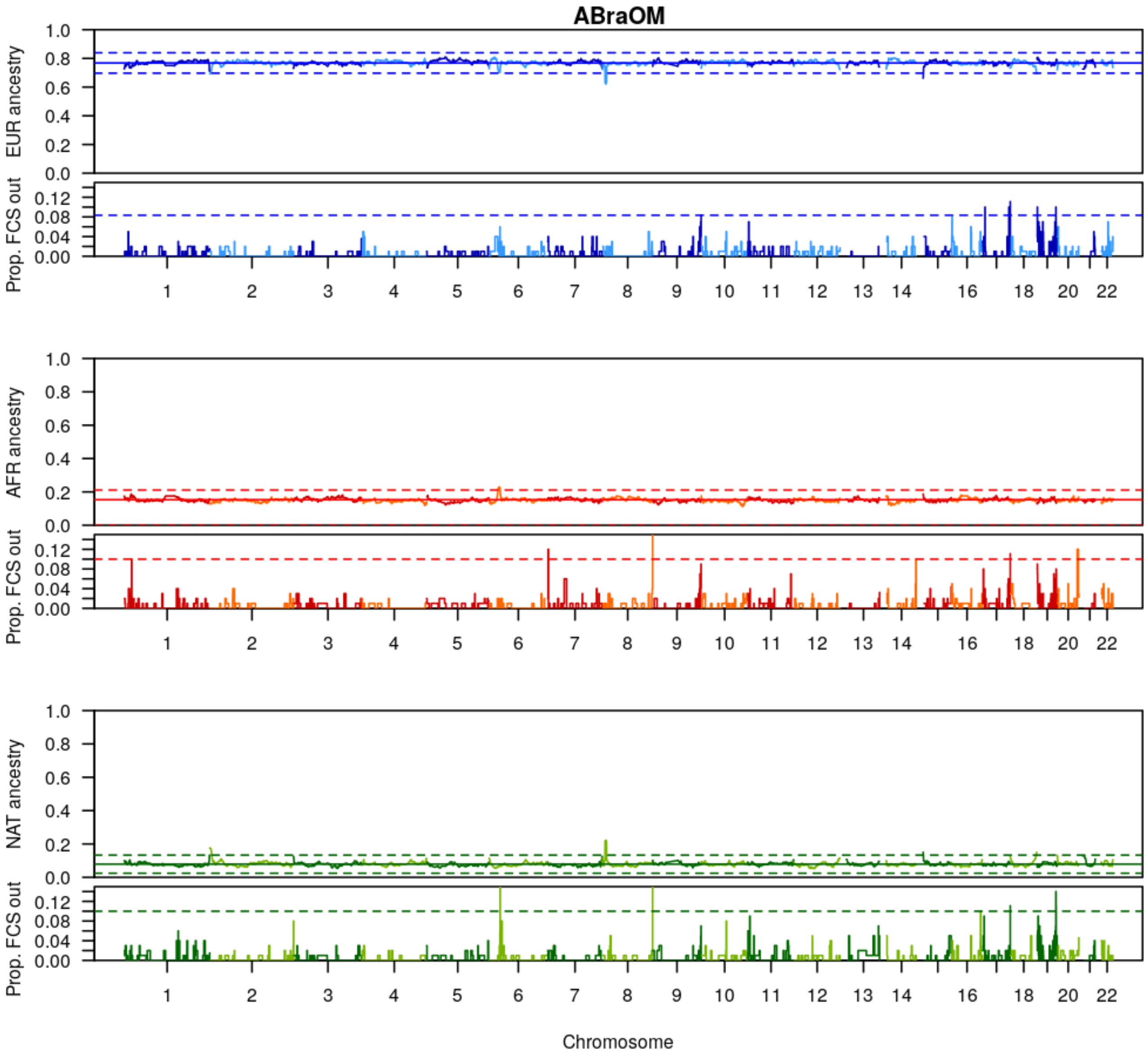

Supplementary Figure 9. Local ancestry inference and signals of positive selection based on Secolin et al., 2019, for the ABraOM exome sample. Each chromosome is shown on the x-axis, and the proportions of European (EUR; grades of blue), sub-Saharan African (AFR; grades of red), and Native-American ancestries (NAT, grades of green) are indicated on the y-axis. Each dashed line indicates a 4.42 SD threshold. The signal of positive selection shows the proportion of FCS outliers (Prop. FCS out) in the y-axis, following the same colour pattern for each ancestry component. Each dashed line indicates the 99.5th percentile thresholds.

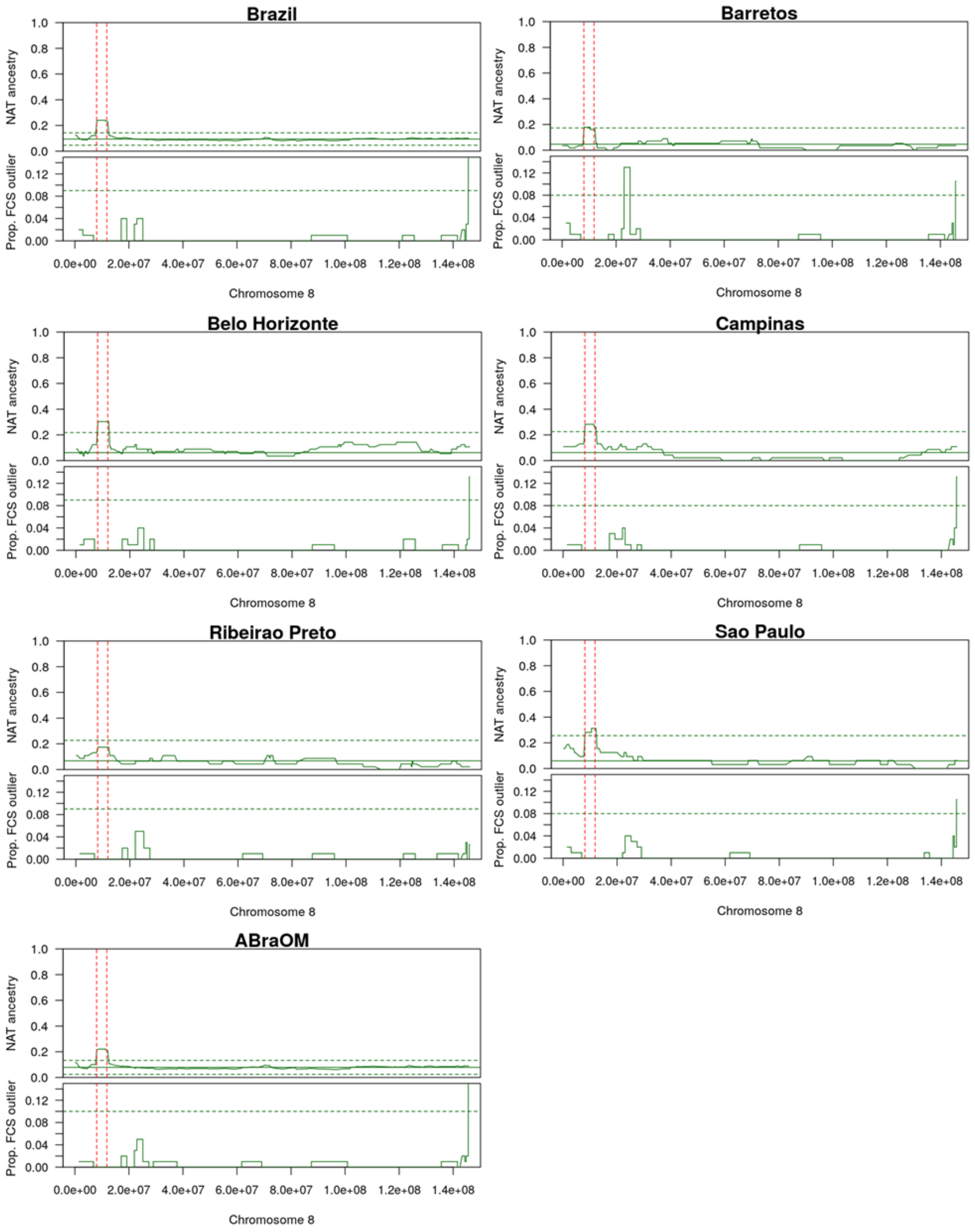

Supplementary Figure 10. Zoom of chromosome 8, showing the Native-American proportion and the proportion of FCS outliers among the Brazilian samples. The ch 8p23.1 region is indicated by the dashed vertical red lines.

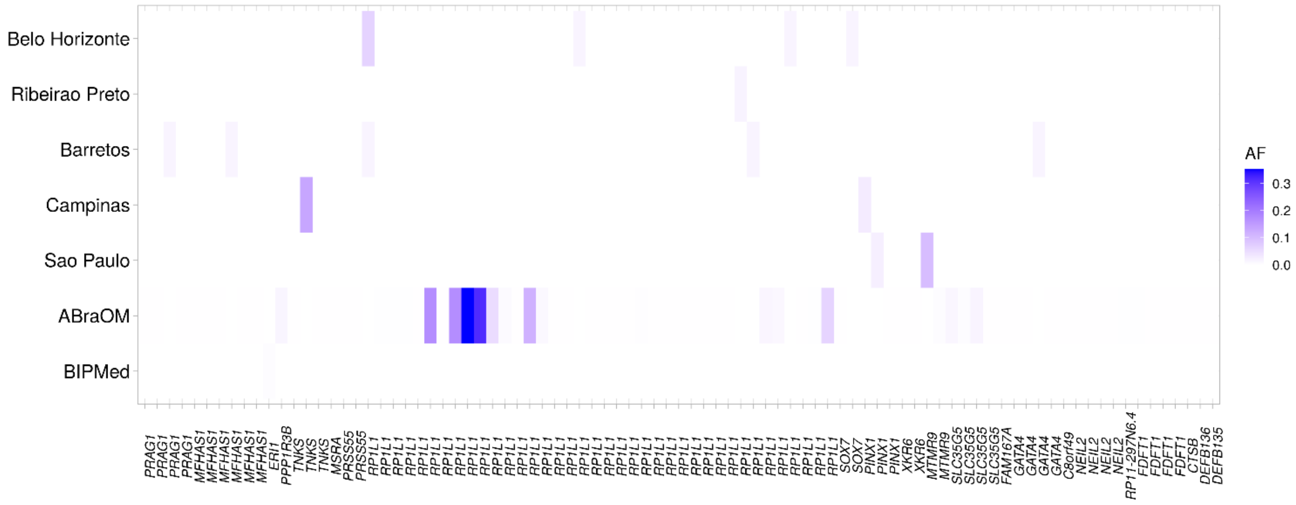

Supplementary Figure 11. Heatmap of the allele frequencies (AFs) of variants found exclusively in the Brazilian population on ch 8p23.1, classified by place of birth and dataset (y-axis). Gene names are shown in the x-axis.

Supplementary Table 1. Percentage of matched ch 8p23.1 inversion between SNP array data (Secolin et al. 2019) and exome sequencing, including 1KGP dataset.

Population	Percentage of inversion match
BIPMed	48.9%
ACB	65.6%
ASW	82.0%
CEU	96.0%
CHB	16.5%
CLM	81.7%
ESN	62.2%
FIN	87.9%
GBR	94.5%
GIH	39.8%
GWD	50.4%
IBS	90.7%
ITU	42.2%
KHV	6.1%
LWK	56.6%
MSL	58.8%
MXL	29.7%
PEL	22.4%
PUR	89.4%
STU	51.0%
TSI	91.6%
YRI	56.5%

Supplementary Table 2. Number of benign and deleterious predictions based on the percentage of concordance among the different algorithms.

Percentage algorithm concordance	Benign predictions	Deleterious predictions
0.0	10	40
0.1	28	130
0.2	30	139
0.3	18	27
0.4	40	17
0.5	26	13
0.6	27	17
0.7	22	12
0.8	123	12
0.9	78	5
1.0	12	2
